# Supplementary material for: The Association between Sugar-Sweetened Beverages and High-Energy Diets and Academic Performance in Junior School Students
Source: Nutrients. 2022 Aug 30;14(17):3577. doi: 10.3390/nu14173577 (PMC9460257; doi:10.3390/nu14173577)
Supplement: Supplementary file 1 [file nutrients-14-03577-s001.zip › nutrients-1857463-supplementary.pdf]

**Table S1.** Association of SSBs and high-energy diet with total academic scores, Chinese, Math, and English scores from the adjusting GLMM Analyses. (Model I and Model II).

| Subjects | Models | Variables | | $\beta$ | 95%CI | $p$-value |
|---|---|---|---|---|---|---|
| Total scores | Model I [a] | SSBs | Seldom (reference) | 0.000 | − | − |
| | | | Sometimes | −1.610 | (−4.513, 1.293) | 0.277 |
| | | | Often | −5.797 | (−9.486, −2.108) | 0.002 |
| | | High-energy diet | Seldom (reference) | 0.000 | − | − |
| | | | Sometimes | 6.909 | (4.107, 9.711) | <0.001 |
| | | | Often | −0.706 | (−4.806, 3.393) | 0.736 |
| | Model II [b] | SSBs | Seldom (reference) | 0.000 | − | − |
| | | | Sometimes | −2.053 | (−4.634, 0.528) | 0.119 |
| | | | Often | −3.198 | (−6.512, −0.116) | 0.059 |
| | | High-energy diet | Seldom (reference) | 0.000 | − | − |
| | | | Sometimes | 2.721 | (0.224, 5.218) | 0.033 |
| | | | Often | −2.145 | (−5.812, 1.522) | 0.252 |
| Chinese | Model I [a] | SSBs | Seldom (reference) | 0.000 | − | − |
| | | | Sometimes | −1.610 | (−4.513, 1.293) | 0.277 |
| | | | Often | −5.797 | (−9.486, −2.108) | 0.002 |
| | | High-energy diet | Seldom (reference) | 0.000 | − | − |
| | | | Sometimes | 6.909 | (4.107, 9.711) | <0.001 |
| | | | Often | −0.706 | (−4.806, 3.393) | 0.736 |
| | Model II [b] | SSBs | Seldom (reference) | 0.000 | − | − |
| | | | Sometimes | −2.053 | (−4.634, 0.528) | 0.119 |
| | | | Often | −3.198 | (−6.512, −0.116) | 0.059 |
| | | High-energy diet | Seldom (reference) | 0.000 | — | — |
| | | | Sometimes | 1.245 | (0.589, 1.901) | <0.001 |
| | | | Often | 0.337 | (−0.627, 1.300) | 0.493 |
| Math | Model I [a] | SSBs | Seldom (reference) | 0.000 | — | — |
| | | | Sometimes | −0.731 | (−2.014, 0.553) | 0.264 |
| | | | Often | −2.064 | (−3.695, −0.433) | 0.013 |
| | | High-energy diet | Seldom (reference) | 0.000 | — | — |
| | | | Sometimes | 1.312 | (0.073, 2.551) | 0.038 |
| | | | Often | −2.301 | (−4.113, −0.488) | 0.013 |
| | Model II [b] | SSBs | Seldom (reference) | 0.000 | — | — |
| | | | Sometimes | −0.959 | (−2.156, 0.238) | 0.116 |
| | | | Often | −1.360 | (−2.897, 0.177) | 0.083 |
| | | High-energy diet | Seldom (reference) | 0.000 | — | — |
| | | | Sometimes | 0.264 | (−0.895, 1.422) | 0.656 |
| | | | Often | −2.071 | (−3.772, −0.370) | 0.017 |
| English | Model I [a] | SSBs | Seldom (reference) | 0.000 | — | — |
| | | | Sometimes | −0.610 | (−1.810, 0.591) | 0.319 |
| | | | Often | −2.672 | (−4.197, −1.147) | 0.001 |
| | | High-energy diet | Seldom (reference) | 0.000 | — | — |
| | | | Sometimes | 3.274 | (2.116, 4.433) | <0.001 |
| | | | Often | 0.438 | (−1.257, 2.132) | 0.613 |
| | Model II [b] | SSBs | Seldom (reference) | 0.000 | — | — |
| | | | Sometimes | −0.753 | (−1.804, 0.299) | 0.160 |
| | | | Often | −1.498 | (−2.848, −0.148) | 0.030 |

| | | | | |
|---|---|---|---|---|
| High-energy diet | Seldom (reference) | 0.000 | — | — |
| | Sometimes | 1.213 | (0.196, 2.230) | 0.019 |
| | Often | −0.411 | (−1.905, 1.083) | 0.590 |

[a] Model I：Non-adjusted model (independent variables include SSBs and high-energy diets). [b] Model II：Model 1+ Fixed effects (gender, household registration, on campus residence, socioeconomic level, parental education level, desk, book collection, internet & computer, TV watching time, internet time, sleep time, and exercise time).
